# Guidelines for the medical management of pediatric vesicoureteral reflux

**DOI:** 10.1111/iju.14223

**Published:** 2020-04-01

**Authors:** Hideshi Miyakita, Yutaro Hayashi, Takahiko Mitsui, Manabu Okawada, Yoshiaki Kinoshita, Takahisa Kimata, Yasuhiro Koikawa, Kiyohide Sakai, Hiroyuki Satoh, Masatoshi Tokunaga, Yasuyuki Naitoh, Fumio Niimura, Hirofumi Matsuoka, Kentaro Mizuno, Kazunari Kaneko, Masayuki Kubota

**Affiliations:** ^1^ Committee for the Formulation of Medical Management Guidelines for Pediatric Vesicoureteral Reflex Japanese Society of Pediatric Urology Osaka Japan; ^2^ Department of Urology Tokai University Oiso Hospital Oiso Kanagawa Japan; ^3^ Department of Pediatric Urology Nagoya City University Graduate School of Medical Sciences Nagoya Aichi Japan; ^4^ Department of Urology University of Yamanashi Graduate School of Medical Sciences Chuo Yamanashi Japan; ^5^ Department of Pediatric General and Urogenital Surgery Juntendo University Hospital Tokyo Japan; ^6^ Department of Pediatric Surgery Graduate School of Medical and Dental Sciences Niigata University Niigata Japan; ^7^ Department of Pediatrics Kansai Medical University Hirakata Osaka Japan; ^8^ Department of Urology Fukuoka City Medical Center of Sick Children Fukuoka Japan; ^9^ Department of Urology Miyagi Children’s Hospital Sendai Miyagi Japan; ^10^ Department of Urology and Kidney Transplantation Tokyo Metropolitan Children’s Medical Center Tokyo Japan; ^11^ Department of Urology Kyoto Prefectural University of Medicine Kyoto Japan; ^12^ Department of Pediatrics Tokai University School of Medicine Hiratsuka Kanagawa Japan; ^13^ Department of Urology Faculty of Medicine Fukuoka University Fukuoka Japan

**Keywords:** medical management guidelines, vesicoureteral reflux

## Abstract

Urinary tract infection is a bacterial infection that commonly occurs in children. Vesicoureteral reflux is a major underlying precursor condition of urinary tract infection, and an important disorder in the field of pediatric urology. Vesicoureteral reflux is sometimes diagnosed postnatally in infants with fetal hydronephrosis diagnosed antenatally. Opinions vary regarding the diagnosis and treatment of vesicoureteral reflux, and diagnostic procedures remain debatable. In terms of medical interventions, options include either follow‐up observation in the hope of possible spontaneous resolution of vesicoureteral reflux with growth/development or provision of continuous antibiotic prophylaxis based on patient characteristics (age, presence/absence of febrile urinary tract infection, lower urinary tract dysfunction and constipation). Furthermore, there are various surgical procedures with different indications and rationales. These guidelines, formulated and issued by the Japanese Society of Pediatric Urology to assist medical management of pediatric vesicoureteral reflux, cover the following: epidemiology, clinical practice algorithm for vesicoureteral reflux, syndromes (dysuria with vesicoureteral reflux, and bladder and rectal dysfunction with vesicoureteral reflux), diagnosis, treatment (medical and surgical), secondary vesicoureteral reflux, long‐term prognosis and reflux nephropathy. They also provide the definition of bladder and bowel dysfunction, previously unavailable despite their close association with vesicoureteral reflux, and show the usefulness of diagnostic tests, continuous antibiotic prophylaxis and surgical intervention using site markings.

**Abbreviations & Acronyms**
BBD: bladder and bowel dysfunction
CAKUT: congenital anomalies of the kidney and urinary tract
CAP: continuous antibiotic prophylaxis
DMSA: dimercaptosuccinic acid
fUTI: febrile urinary tract infection
RN: reflux nephropathy
SFU: Society for Fetal Urology
UTI: urinary tract infection
VCUG: voiding cystourethrography
VUR: vesicoureteral reflux

## Introduction

UTI is a form of bacterial infection that occurs commonly in children. VUR is a major underlying precursor condition of UTI, and an important disorder in the field of pediatric urology. VUR is sometimes diagnosed postnatally in patients with fetal hydronephrosis diagnosed antenatally. Although opinions vary regarding the diagnosis and treatment of VUR, diagnostic procedures are still debatable. These guidelines, formulated and issued by the Japanese Society of Pediatric Urology to assist in the medical management of VUR consistent with the clinical practice algorithm, explain the epidemiology and the following elements: syndromes (dysuria with VUR, and bladder and rectal dysfunction with VUR), diagnosis, treatment (medical and surgical), secondary VUR, long‐term prognosis and RN.

### 1 Concept of VUR

#### (1) Definition

VUR is a condition characterized by retrograde flow of urine accumulated in the bladder back to the renal calyx or to the kidney (intra‐renal reflux) through one or both ureters because of immaturity of the preventive mechanism against reflux or failure as a result of anatomical or functional abnormalities.

#### (2) Classification of VUR

Primary VUR develops as a result of an impaired or immature preventive mechanism against reflux due to anatomical or functional congenital abnormalities. Secondary VUR is that due to a defect of this preventive mechanism from organic obstruction and/or neurological dysfunction (posterior urethral valve, anterior urethral diverticulum, urethral hypoplasia and neurogenic bladder) in the lower urinary tract.^1^, ^2^

### 2 Epidemiology of VUR

#### (1) Incidence of VUR and settings of incidental diagnosis

The estimated incidence of VUR is approximately 1% (0.4–1.8%), but the precise figure, including asymptomatic cases, is unknown.^3^ An analysis of 774 pediatric patients (infants aged <1 year and children aged ≥1 year) with grade IV–V VUR showed that VUR was detected in the following settings: examination for UTI (88%), examination for symptoms of BBD (4%), thorough examination for familial VUR (7.4%) and postnatal examination for fetal hydronephrosis (1%).^4^

#### (2) Incidence of VUR detected on examination for fetal hydronephrosis

VUR was detected in 16.2% (7–35%) of patients with hydronephrosis diagnosed using fetal ultrasound. Among them, the majority were boys (70%), and one‐third had high‐grade reflux (grade IV–V).^5^ Renal damage was found in 6.2% of patients with grades I–III VUR, and in 47.9% of those with grade IV–V VUR. Renal damage was also found in 21.8% of patients without UTI (presumed congenital RN).^5^

#### (3) Incidence of VUR detected on examination for familial VUR

The incidence of VUR is higher in children with a positive family history. According to a meta‐analysis, VUR was detected in 27.4% (3–51%) of siblings of children with VUR, and 35.7% (21.2–61.4%) of offspring of parents with VUR; many had mild VUR (grade I–II), and 9.8% had grade III–V VUR.^5^

#### (4) Incidence of VUR in UTI

VUR occurs in 36–56% of children with UTI, and the detection rate increases with earlier age of onset of UTI; VUR was detected in 70%, 25%, 15% and 5.2% of UTI patients if onset was in infancy, at age 4 years, at age 12 years and in adulthood, respectively.^1^ Also, the frequency of renal damage increased with increased frequency of UTI,^4^, ^6^, ^7^, ^8^, ^9^, ^10^, ^11^ and renal scarring was found in 26%, 38% and 80% of patients who had no recurrence of UTI, had one recurrence and had two or more recurrences, respectively.^10^

### 3 Incidence of VUR in CAKUT

VUR occurs at high rates of 46.0% as a common complication of CAKUT in patients with duplicated ureter, and 16.1% in those with pelviureteric junction obstruction.^12^ A meta‐analysis showed that 24% of 770 patients with unilateral renal agenesis had VUR.^13^

### 4 VUR and genetic inheritance

Many patients with VUR have familial VUR with an 80–100% concordance in monozygotic twins and 35–50% concordance in dizygotic twins. Because of the high concordance rate for VUR in monozygotic twins, genetic anomalies are thought to be a causative factor of VUR. VUR of genetic origin is thought to be inherited in an autosomal dominant manner.^14^

There are no specific genes proven to be responsible for sporadic non‐syndromic VUR compared with syndromic VUR. Candidate genes responsible for VUR as a complication of CAKUT include *PAX2* (renal coloboma syndrome), *EYA1* (branchio‐oto‐renal syndrome), *GREM1*, *UPK3A*, *UPK2*, *HNF1B*, *ROBO2*, *SIX2*, *BMP4*, *SOX17* and *TNXB*.^15^

Angiotensin type 2 receptor knockout mice showed familial developmental anomalies of the kidney and urinary tract, including VUR.^16^ Thus, VUR is presumed to be a part of the sequential developmental anomalies of the kidney and urinary tract caused by genetic abnormalities.

**Fig. 1:**
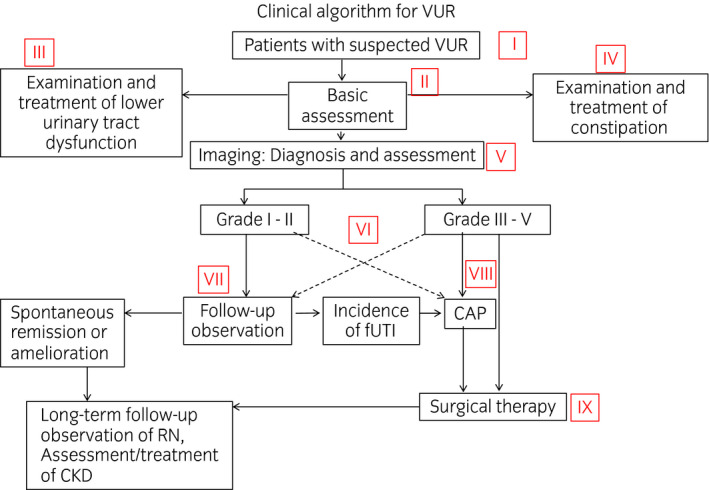
Clinical algorithm for VUR.

These guidelines explain the ideal clinical practice for VUR using a novel algorithm (Fig. 1).

## I Settings of diagnosis (patients with suspected VUR)

VUR is often diagnosed in patients with fUTI,^4^, ^15^ and because of VUR diagnosed at screening in infants with fetal hydronephrosis detected by ultrasound, infant health checks using ultrasound and thorough examination for familial VUR are also on the rise.^5^, ^6^ In patients with BBD, VUR is detected during examination for voiding and/or storage symptoms. In patients with advanced renal dysfunction, VUR is detected during school health checks that reveal proteinuria or symptoms of kidney failure.

## II Basic assessment

History taking, physical examination, urine tests and ultrasound must be included in the basic assessment. A thorough family history is particularly important because of the possibility of familial VUR. Fever is often the only apparent symptom of UTI in young patients, and they might have complaints of urgency, frequency, dysuria, and abdominal pain only after they have completed toilet training and are able to talk. Midstream urine can be collected if the child can express the urge to urinate, but clean‐catch urine will be collected using a urine collection pack for younger children irrespective of sex. UTI will be diagnosed based on pyuria (urinary white blood cell count ≥10/high‐power field) and/or bacteriuria. A bacterial count ≥10^5^ cfu/mL, detected by culture, reliably identifies the causative organism(s).

Ultrasound findings of upper urinary tract dilatation indicate possible VUR, and hyperplasia or abnormal morphology of the bladder wall suggest voiding dysfunction associated with VUR. Thus, in patients with SFU grade 3 or 4 hydronephrosis with upper urinary tract dilatation, the presence of VUR should be determined using VCUG. However, if postnatal ultrasound does not show hydronephrosis, serious malformation of the urinary tract is unlikely, although the possibility of VUR cannot be fully excluded. In infants with fetal hydronephrosis diagnosed antenatally, the presence of fUTI should raise suspicion for VCUG, even though hydronephrosis has resolved.^5^, ^16^

## III Thorough examination and treatment of lower urinary tract dysfunction

Lower urinary tract symptoms (e.g. incontinence, urgency, frequency, dysuria, hesitancy and straining), and/or abdominal and intestinal abnormalities (e.g. constipation and encopresis) are often observed.^17^, ^18^

### 1 Lower urinary tract dysfunction and VUR

#### (1) Before completion of toilet training

It is suggested that lower urinary tract dysfunction characterized by increased residual volume and bladder capacity is involved in the occurrence of VUR before completion of toilet training.^19^

#### After completion of toilet training

Lower urinary tract dysfunction, typified by dysfunctional voiding, overactive bladder (detrusor overactivity) and infrequent voiding, is closely associated with VUR after completion of toilet training.^20^, ^21^

#### (2) Involvement of lower urinary tract dysfunction in spontaneous resolution of VUR

In VUR complicated by lower urinary tract dysfunction, the possibility of amelioration or resolution of VUR is low.^22^, ^23^, ^24^, ^25^ Also, for patients with lower urinary tract dysfunction, such as detrusor overactivity and infrequent voiding, spontaneous resolution of VUR took longer to ensue after conservative treatment.^20^

#### (3) Resolution or amelioration of VUR after treatment of lower urinary tract dysfunction

Treatment of lower urinary tract dysfunction, particularly dysfunctional voiding, was effective for resolution of VUR.^26^ Also, the rate of resolution or amelioration of VUR was higher in children who received biofeedback treatment, where patients undergo training after they are able to perceive abnormal physiological responses based on the information on the contraction of the urethral sphincter and pelvic floor muscle represented by sounds and graphics, than those who did not.^27^ Anticholinergic medication was as effective as surgical treatment in resolving or ameliorating VUR in children with detrusor overactivity, and thus conservative management using anticholinergic medication is recommended.^28^

## IV Constipation

### IV.1 BBD and VUR

The frequency of bowel movement ≤2/week, recurring absence of bowel movement for ≥5 days, frequent but small quantity of feces or fecal impaction in the rectum (size of a hen’s egg or larger) detected by abdominal X‐ray/ultrasound were associated with VUR with BBD,^29^, ^30^, ^31^ and thus thorough examination and treatment by specialists is required.

In terms of the age at which voiding and bladder function are established, we propose a new definition of BBD.

#### IV.1.1 Definition

BBD is a condition where either of the following exist: (i) a symptom of lower urinary tract dysfunction**,** which is basically identified with objective assessment tools, such as uroflowmetry or voiding diary, without clear organic factors (neurogenic bladder, external injury and congenital malformation) in patients aged ≥5 years who have achieved toileting independence; and/or (ii) an abnormal abdominal and intestinal finding (frequency of bowel movement ≤2/week, recurring absence of bowel movement for ≥5 days, frequent but small quantity of feces or fecal impaction [size of a hen’s egg or larger] in the rectum detected using abdominal X‐ray/ultrasound) in accordance with the Clinical Practice Guidelines for Pediatric Chronic Functional Constipation by the Japanese Society for Pediatric Gastroenterology, Hepatology and Nutrition.^32^

## V Imaging: Diagnosis and assessment

Renal and bladder ultrasound, VCUG, and ^99m^Tc‐DMSA renal scintigraphy are used.

1. Renal and bladder ultrasound: Urinary tract malformation, such as hydronephrosis, hypoplastic kidney, duplicated ureter, megaloureter and ureterocele, can be accurately diagnosed. This modality is useful because of its low invasiveness and ease of repeated use. Ultrasound findings, such as pyelectasis, abnormal echogenicity, differences in renal dimensions between the right and left kidney, irregular contour, ureterocele, and dilatation of the lower ureters at the dorsal aspect of the bladder, are important, because they are possible risk factors for UTI. Ultrasound screening did not detect any abnormalities in 46–60% of patients with VUR detected by VCUG.^33^, ^34^, ^35^ The accuracy of diagnosis of VUR based on the presence/absence of ultrasound abnormalities is low, with a sensitivity of 18–46%, specificity of 76–88%, positive predictive value of 24–66% and negative predictive value of 71–83%.^33^, ^34^, ^35^
2. VCUG: VCUG is a standard imaging test used for the diagnosis of VUR. It offers detailed anatomical information, enabling determining the presence/absence of VUR, and also the severity grade according to the international classification.^36^, ^37^, ^38^ VCUG is required for assessment of the lower urinary tract, as well as diagnosis of VUR.^20^, ^23^, ^28^, ^39^
3. ^99m^Tc‐DMSA renal scintigraphy: ^99m^Tc‐DMSA renal scintigraphy is a standard imaging test used for diagnosis of renal parenchymal damage, and is suited for assessment of split renal function and renal scarring in patients with VUR.

### V.1 Settings of diagnosis of VUR

#### 1 Screening of infants with fetal hydronephrosis diagnosed antenatally using fetal ultrasound

Renal and bladder ultrasound is the standard test for newborns with fetal hydronephrosis diagnosed antenatally.^40^, ^41^, ^42^ VCUG is necessary to confirm the presence/absence of VUR in patients with SFU grade 3 or 4 hydronephrosis with ureteral dilatation.

#### Screening after fUTI

##### (1) First episodes of fUTI

Approximately 30–50% of young children with fUTI have VUR, and thus there is no clear evidence to rule out the fact that all patients with a first episode of fUTI should undergo VCUG. VCUG is also useful for assessment of functional and organic abnormalities of the lower urinary tract, and some patients with the first episode of fUTI need to be examined with VCUG.^20^, ^23^, ^28^, ^43^

##### (2) Recurrent episodes of fUTI

VCUG should be carried out in patients with recurrent episodes of fUTI, because they are highly likely to have VUR. Also, given that fUTI is a precondition for renal scarring, fUTI recurrence is a risk factor. Thus, patients with recurrent episodes of fUTI need to be examined for renal scarring using ^99m^Tc‐DMSA renal scintigraphy, as well as for VUR using VCUG.

##### (3) Screening with a focus on renal parenchymal lesions rather than the presence/absence of VUR

Changes in fUTI management strategies in young children are based on the viewpoint that the presence/absence of renal parenchymal lesions, rather than VUR, determines renal functional outcomes. Inevitably, VUR is more likely to be missed, but the most undetected cases were reportedly of mild VUR that did not cause renal scarring.^44^, ^45^, ^46^, ^47^ Also, in high‐grade VUR (international VUR classification grade III–V), the frequency of breakthrough UTI is overwhelmingly higher in patients with renal scarring (60%) than in those without scarring (6%), and thus assessment of renal scarring by ^99m^Tc‐DMSA renal scintigraphy is useful.^48^

The level of usefulness of each test for diagnosis of pediatric VUR is shown in Table 1.

**Table 1:** Diagnosis of pediatric VUR: level of usefulness of each test

		Ultrasound	VCUG	DMSA renal scintigraphy
Hydronephrosis detected using fetal ultrasound	Medical history of fUTI (−)	★★★	▲ (★★; SFU grade 3–4, with ureteral dilatation)	▲
	Medical history of fUTI (+)		★★★	★
After fUTI	First episode	★★★	★★	★
	Recurrent episodes		★★★	★★★
Lower urinary tract abnormalities (suspected)	Medical history of fUTI (+)	★★★	★★★	★

★★★, Considered as standard; ★★, considered as standard depending on the condition; ★, considered as optional; ▲, not recommended.

## VI Diagnosis of VUR and assessment of renal scarring in relation to VUR grades

For children who have not achieved toileting independence (from the neonatal period to the completion of toilet training): if patients do not have fUTI, follow‐up observation with optional CAP is recommended for grade I–II VUR, whereas CAP is recommended for grade III or severe VUR; and CAP should be considered in patients with fUTI. For children who have completed toilet training, follow‐up observation is recommended for grade I–II VUR without cortical abnormalities, whereas CAP is recommended for grade III–V VUR with cortical abnormalities. Follow‐up observation will be considered even for grade III–V VUR if there is no renal scarring without fUTI. Please see the section of “conservative therapy” for details.

## VII Follow‐up observation

The rate of spontaneous resolution of VUR in infants within 1–4 years was 50%, with a slightly higher rate for girls; the rate was 71% if VUR was of grade I–III, and 28% if VUR was of grade IV–V; the resolution rate was high in infants, and VUR was resolved at a yearly rate of 9% after infancy.^49^ Also, follow up for 2 years on average showed a spontaneous resolution rate of 51% (72% for grade I, 61% for grade II, 49% for grade III and 32% for grade IV–V), and that the resolution rate was higher when the VUR grade was lower, VUR was bilateral (compared with unilateral), patients were younger and VUR was detected on screening for patients with fetal hydronephrosis or suspected familial VUR.^50^

CAP should be considered when VUR does not resolve spontaneously.

## VIII CAP

CAP is widely accepted as a revolutionary conservative therapy for VUR, because it prevents recurrent episodes of fUTI and consequent new renal scarring. The Randomized Intervention for Children with Vesicoureteral Reflux trial showed that CAP using a combination of trimethoprim and sulfamethoxazole reduced the occurrence of UTI by approximately half (from 27.4% to 14.8%) in patients aged 2–71 months with VUR diagnosed after the first UTI, but did not prevent new renal scarring. It is noteworthy that the presence of antibiotic‐resistant bacteria in cases of recurrent episodes of fUTI was significantly higher in the CAP‐treated group than the CAP‐untreated group (68.4% *vs* 24.6%). Surgical therapy is to be considered for breakthrough UTI (CAP is judged ineffective in such cases).

The choice between CAP and follow‐up observation is to be made based on patient characteristics, including age, and the presence/absence of fUTI, lower urinary tract dysfunction and constipation. Lower urinary tract dysfunction and constipation should be treated accordingly.

### 1 Indications for CAP

Given that BBD becomes apparent after completion of toilet training, here, we discuss the indications for CAP in two phases – before and after completion of toilet training (establishment of voluntary micturition); BBD is only taken into account in the latter phase.

#### (1) From birth to the completion of toilet training

##### (a) Patients without fUTI

These rare cases include those of VUR detected using VCUG based on findings of fetal and neonatal ultrasound. According to the consensus statement issued in 2010 by the SFU, it is recommended that CAP be started before confirmation of VUR if SFU grade is ≥3, and be continued if VUR is confirmed. If fUTI is absent, these guidelines recommend CAP for grade III or severe VUR, but CAP is optional for grade I–II VUR.^51^

##### (b) Patients with fUTI

CAP is recommended irrespective of the severity of VUR if fUTI is present, especially in patients aged <1 year. In patients aged ≥1 year, it is difficult to detect BBD before completion of toilet training. Taken together, these guidelines recommend CAP if fUTI is present for patients with grade I–V VUR who have not completed toilet training.

#### (2) After completion of toilet training

##### (a) Patients without fUTI and BBD

Pediatric patients with these characteristics are rare, and the likelihood of encountering such patients is low.

In these guidelines, CAP is recommended for patients with grade II–V VUR with renal cortical abnormalities, otherwise CAP is optional.

##### (b) Patients without fUTI, but with BBD

BBD must be treated. Given that the risk of fUTI is high in patients with BBD, and CAP is expected to be effective in those patients, CAP is recommended for grade III–V VUR.

##### (c) Patients with fUTI, but without BBD

For grade III or severe VUR, CAP is the second choice after surgical intervention, but is recommended only until implementation of surgical intervention or if surgery is not selected for some reason. For grade I–II VUR, CAP is optional.

##### (d) Patients with fUTI and BBD

CAP is recommended for grade I–V VUR with the precondition that the utmost priority is treatment of BBD, and if surgery is indicated, surgical intervention will be chosen as the preferred option.

### 2 Antimicrobials for CAP

Susceptibility of intestinal bacteria to antimicrobials and the possibility of emergence of antimicrobial resistance should be considered when choosing antimicrobials for CAP. Cephem antibiotics, penicillin antibiotics, and combinations of trimethoprim and sulfamethoxazole are widely used in the clinical setting. Combinations of trimethoprim and sulfamethoxazole were used mainly in recent clinical studies on the usefulness of CAP in Europe and North America.

Cephem antibiotics (especially relatively traditional agents) are widely used in Japan, and can be used from early infancy.^52^, ^53^ A single oral dose (between one‐third and one‐sixth of the normal pediatric daily dose) should be given before bed.

Penicillin antibiotics can be administered to infants aged <2 months; a single oral dose (one‐third of the pediatric daily dose) should be given before bed.

Trimethoprim and sulfamethoxazole, which are used in combination in many clinical studies, are considered standard medication. However, they should not be administered to infants aged <2 months; thrombotic thrombocytopenic purpura and hemolytic–uremic syndrome are listed as serious adverse reactions on the package insert, and adequate observation by blood tests is necessary.

### 3 Timing of discontinuation of CAP

At present, there is no clear consensus on the timing of discontinuation of CAP, and the following options are considered depending on age (months and years) at the start of CAP: (i) at completion of toilet training; (ii) after CAP for 1–2 years, even if toilet training has not yet been completed; (iii) at confirmation of spontaneous resolution of VUR; and (iv) at new onset or recurrence of breakthrough UTI (when CAP is deemed ineffective). The validity of discontinuing CAP after the completion of toilet training was shown in a relatively large number of studies.^54^, ^55^, ^56^, ^57^, ^58^, ^59^, ^60^ Also, it was reported that CAP discontinuation becomes an option even before completion of toilet training if patients have residual VUR of grade I–II without recurrent episodes of fUTI during follow up.^61^

The level of usefulness of CAP in treatment of pediatric VUR is shown in Table 2.

**Table 2:** Level of usefulness of CAP for pediatric VUR

Age	Presence or absence of fUTI	Presence or absence of BBD	VUR grade	Usefulness level
From birth to completion of toilet training	fUTI (−)	BBD‐unknown	I	★ ★★
			II	
			III	
			IV	
			V	
	fUTI (+)	BBD‐unknown	I	★★
			II	
			III	
			IV	
			V	
After completion of toilet training	fUTI (−)	BBD (−)	I	★
			II	★ (★★; cortical abnormalities)
			III	
			IV	
			V	
		BBD (+)	I	★
			II	
			III	★★
			IV	
			V	
	fUTI (+)	BBD (−)	I	★★
			II	
			III	
			IV	
			V	
		BBD (+)	I	
			II	
			III	
			IV	
			V	

★★, Considered as standard depending on the condition; ★, considered as optional.

### 4 Treatment of BBD

1. Behavioral therapy: Patients are encouraged to void the bladder and bowel regularly. This therapy helps patients acquire regular voiding habit, thereby improving control of the lower urinary tract and bowel. Thus, this therapy serves as the basis of all other therapies.
2. Pharmacotherapy: Oral therapy using mainly anticholinergics and α‐blockers are predominantly used for storage symptoms (e.g. urge incontinence and frequency) and voiding symptoms.
3. Biofeedback therapy: This is indicated for children aged ≥5 years. Devices are used to provide visual, tactile and other sensory information to help patients understand the contraction of the pelvic floor muscles, and such understanding will be used to improve lower urinary tract function.
4. Treatment of defecation problems: Correction of fluid intake, dietary intervention and use of laxatives.
5. Urotherapy:^62^, ^63^ Comprehensive conservative therapy by specialists is necessary, including: (i) good understanding of the anatomy and physiology of the lower urinary tract; (ii) advising patients on how to alleviate lower urinary tract dysfunction (e.g. appropriate urination posture, regular urination and not holding urine); (iii) advice on lifestyle (e.g. fluid intake and diet); and (iv) psychological approaches for patients with BBD.

## IX Surgical therapy

Indications: Current indications, albeit controversial, include: (i) breakthrough UTI and poorly controlled UTI; (ii) high‐grade VUR; (iii) renal impairment (at detection or during follow‐up observation); (iv) multiple UTI recurrences in patients older than those for whom CAP is indicated; and (v) high‐grade VUR with lower urinary tract dysfunction.

### 1 Endoscopic injection

The rate of resolution of reflux after endoscopic injection is lower than those after open surgery and laparoscopic surgery. Treatment is covered by health insurance for grade II–IV VUR, but not for grade V VUR. Complications include new‐onset contralateral VUR and ureteral obstruction.

#### (1) Maneuvers and indications

This minimally invasive day procedure is a new option in addition to the conventional curative surgery, ureterocystoneostomy, but there is no consensus on selection criteria.

#### (2) Efficacy and safety

A meta‐analysis showed that the rate of the resolution of reflux after the first endoscopic injection was 74%; the rates of resolution of grade II, grade III and grade IV VUR were 79%, 72% and 63%, respectively.^64^

The incidence of UTI was lower after endoscopic injection (15%) than after open surgery (38%), and the incidence of fUTI was also lower after endoscopic injection (5%) than after open surgery (24%).^65^

The rate of VUR resolution after open surgery was unaffected by the presence of BBD, but that after endoscopic injection was significantly reduced with BBD.^17^ Also, the resolution rate was low in VUR complicated with duplicated ureter (50%) and neurogenic bladder (62%).^66^ Endoscopic injection is indicated for residual reflux after open surgery, resulting in resolution rates of 68–83%.^67^, ^68^

### 2 Open surgery for VUR

#### 2.1 Open surgery

This includes many techniques, such as the Cohen, Politano–Leadbetter and Lich–Gregoir techniques. All involve incisions of the skin and bladder, and extension of the ureter in the bladder wall, and can be indicated for all patients who are surgically treatable. The efficacy of open surgery was proven to be high irrespective of the technique used, and it is indicated for patients with malformation and functional abnormalities of the urinary tract (e.g. BBD and duplicated ureter). It is standard surgical therapy with confirmed good long‐term efficacy and low rates of complications.

#### 2.2 Success rates of open surgery

The success rates of open surgery for primary VUR were reported to be very high (95–99%) irrespective of the severity of reflux.^17^ Different therapy strategies are recommended depending on the presence of BBD in recent years; however, unlike endoscopic injection, open surgery offers similar therapeutic outcomes between groups with BBD (success rate 88.4–100%) and those without BBD (success rate 92–100%).^69^, ^70^, ^71^, ^72^

### 3 Laparoscopic surgery for VUR

#### (1) Intravesical approach

##### (a) Indications

The approximate age of 3 years was calculated using a formula that relates age and bladder capacity as: bladder capacity = 25 × (age [years] + 2).^73^ However, this formula estimates bladder capacity without anesthesia, and under anesthesia an insufflated bladder has a slightly larger capacity than estimated and is also more expandable. Taken together, the age of patients treatable by this procedure needs to be decided based on the ability and experience of the surgeon.

##### (b) Efficacy and safety

The laparoscopic transvesical Cohen’s technique has a success rate (91–96%) close to that of open surgery.^74^, ^75^, ^76^, ^77^, ^78^, ^79^, ^80^, ^81^ However, the difficulty of the procedure and relatively long learning curve should be considered to carry out this laparoscopic surgery. Complications include intraperitoneal urinary leakage, anastomotic stricture and hematuria.^74^, ^75^, ^76^ Urine leakage from the ports was reported to have occurred in 12.6% of patients, and anastomotic stricture in 6.3% of patients; as described earlier, the incidence of complications was higher with a smaller bladder capacity and younger patients.^3^ The incidence of anastomotic stricture was shown to be similar between laparoscopic surgery and open surgery.^8^ The Politano–Leadbetter technique has a success rate of 94.4%, and is recommended for treatment in women with high‐grade VUR requiring the creation of a long submucosal tunnel, although with a longer operation time than Cohen’s technique.^82^

#### (2) Extravesical approach

##### (a) Indications

Several studies have reported on laparoscopic surgery using an extravesical approach in patients aged 12 months.^83^, ^84^, ^85^ The important consideration for the age of patients treatable by surgical procedure is whether adequate working space can be achieved. Compared with the intravesical approach, the extravesical approach has a wider working space (i.e. abdominal cavity), and thus the age treatable by the extravesical approach can be younger than the age treatable by the intravesical approach.

##### (b) Efficacy and safety

High success rates (93.5–100%) similar to that of open surgery were reported.^17^, ^83^, ^85^, ^86^, ^87^, ^88^ The complications reported include ureteral injury, intraperitoneal urinary leakage and ureteral stenosis.^17^, ^83^, ^87^, ^88^ The incidence of voiding problems, which are characteristic complications of bilateral surgery using the extravesical approach, was 6.5% after laparoscopic surgery,^83^ and was approximately 3–20% after open surgery.^89^, ^90^

#### (3) Robot‐assisted surgery

Indications for robot‐assisted surgery conform to those for open surgery and laparoscopic surgery, but robot‐assisted surgery is indicated for children weighing ≥10 kg in principle to prevent robotic arm interference. Nevertheless, this procedure is not currently covered by health insurance in Japan.

##### (3).1 Comparison between robot‐assisted surgery and laparoscopic surgery

The rates of reflux resolution did not differ significantly between laparoscopic surgery (92.3%) and robot‐assisted surgery (93.3%), and no serious complications were reported.^91^ The cost of robot‐assisted surgery was reported to be significantly higher than that of laparoscopic surgery,^92^ but Weiss *et al*. proposed that long‐term outcomes be assessed in future, because robot‐assisted surgery is undoubtably a beneficial, safe and non‐invasive option.^93^

The level of usefulness of surgical therapies for VUR is shown in Table 3.

**Table 3:** Level of usefulness of surgical therapies for VUR

Surgery	Surgical technique	Conditions	Level of usefulness
Open surgery	Politano–Leadbetter technique		★★★
	Cohen technique		★★★
	Lich–Gregoir technique	Unilateral	★★★
		Bilateral	★
Laparoscopic surgery	Politano–Leadbetter technique		★
	Cohen technique		★★
	Lich–Gregoir technique	Unilateral	☆☆
		Bilateral	☆☆
Robot‐assisted surgery	Politano–Leadbetter technique		△
	Cohen technique		△
	Lich–Gregoir technique	Unilateral	☆☆
		Bilateral	☆☆
Endoscopic injection	Deflux	Grade I	☆
		Grade II	★★
		Grade III	★★
		Grade IV	★★
		Grade V	☆
		BBD (+)	▲
		Duplicated ureter	★
		Residual reflux after surgery	★★

★★★, Considered as standard; ★★, considered as standard depending on the condition; ★, considered as optional; ▲, not recommended; ☆☆, considered as standard depending on the condition, but not approved or covered by health insurance; ☆, considered as optional, but not approved or covered by health insurance; △, not recommended, and not approved or covered by health insurance.

## X Secondary VUR

Secondary VUR is defined as VUR secondary to functional or organic abnormalities of the urinary tract. Neurogenic functional abnormalities include neurogenic bladder due to spinal cord disorders (e.g. spina bifida) and brain disorders (e.g. cerebral palsy); non‐neurogenic organic abnormalities include posterior urethral valve, ureterocele and phimosis. Table 4 shows diseases associated with secondary VUR.^94^

**Table 4:** Secondary VUR

Neurogenic disorders (neurogenic bladder)	Spinal cord disorders	Spina bifida
		Agenesis or hypoplasia of the sacrum
		Spinal cord tumor
		Traumatic spinal cord injuries
	Brain disorders	Cerebral palsy
		Brain tumor
		Traumatic encephalopathy
Non‐neurogenic organic disorder of urinary tract	Urethral disorders	Posterior urethral valve
		Anterior urethral valve
		Megalourethra
		Duplicated urethra
		Prostatic urethral polyps
		Congenital mental stenosis (in girls)
	Ureteral disorders	Ureterocele
		Ectopic ureteric opening (associated ureterocele)
	Bladder diseases	Congenital bladder neck sclerosis
		Megacystis microcolon intestinal hypoperistalsis syndrome
		Prune berry syndrome
		Bladder exstrophy
	Phimosis	Complete phimosis
Others		Complications of imperforate anus
		Complications of cloacal anomalies
		Pharmaceutical‐induced
		After kidney transplantation

## XI Long‐term prognosis and long‐term follow‐up observation

Studies that examined VUR in pediatric patients with chronic kidney disease showed that intermediate‐to‐severe proteinuria was found in approximately one‐third of patients, and the mean rate of reduction in creatinine clearance was significantly larger in these patients than in patients without proteinuria or with mild proteinuria;^95^ and proteinuria was associated with impairment of renal function.^96^ Overt proteinuria is considered an index of poor prognosis; urinary protein of 0.2–0.5 g/day was associated with an increased risk of progression of renal dysfunction, 0.8–1.0 g/day with an increased risk of end‐stage renal disease.^95^, ^97^ Increased excretion of β2‐microglobulin, retinol‐binding protein, α1‐ microglobulin and *N*‐acetyl‐beta‐D‐glucosaminidase in tubulointerstitial failure was reported.^98^, ^99^, ^100^, ^101^

### XI.1 After conservative therapy and surgical therapy

Conservative therapy and surgical therapy for high‐grade VUR were investigated in several randomized controlled studies. A randomized controlled study examined two groups of patients aged <10 years with grade III–IV VUR for 6–54 months, and found that the incidence of renal scarring was not significantly different between the conservative therapy group (22%) and the surgical therapy group (31%), whereas that of fUTI was 22% in the conservative therapy group and 8% in the surgical therapy group, confirming the benefit of surgical surgery.^102^

## XII Pathology of RN

RN occurs where VUR is accompanied with renal parenchymal damage. The term was proposed by Bailey in 1973, and refers to renal dysmorphism (renal parenchymal damage) found frequently in patients with VUR.^103^

Congenital RN is renal hypo/dysplasia caused by embryological factors, or that develops in the presence of promoting factors, such as a functional obstructive mechanism as a result of reflux or urinary tract obstruction based on urodynamic abnormalities and high‐pressure voiding during fetal life and infancy, especially in boys. The condition is generally thought to be diffusive, spreading out across the entire kidney, but can be segmental in some cases.

Acquired renal parenchymal damage includes renal scarring resulting from acute pyelonephritis. These are identified as fibrotic regions under the microscope, or deformed and/or dilatated kidney calyces and accompanying thinning regions in the renal cortex.

Patients with a large mass of renal parenchymal lesions tend to have a small number of nephrons in the remaining parenchyma, which might cause hyperfiltration and then progressive RN. Impaired renal function, hypertension and proteinuria are important indicators of RN, and are significantly associated with long‐term prognosis of renal function.

### XII.1 Progressive RN

Complications, such as increased serum creatinine levels, impaired renal function (e.g. decreased glomerular filtration rate), proteinuria and/or hypertension, indicate progressive RN. It is generally thought that RN progresses when the remnant normal region mass, a decrease of which negatively correlates with an increase in the damaged region mass in the renal parenchyma, is reduced to the level below threshold, but details, such as marginal regions, remain unknown.^104^ Many patients with progressive RN have bilateral multiple renal parenchymal injury.^105^

## Conflict of interest

None declared.
